# A Statistical Account of Fractures, Treated in the Pennsylvania Hospital, from Its Foundation, in 1751, to 1800, with Remarks on the Mode of Treatment, &c.

**Published:** 1838-01-17

**Authors:** J. M. Wallace

**Affiliations:** Resident Surgeon


### A STATISTICAL ACCOUNT OF FRACTURES,

*Treated in the Pennsylvania Hospital, from its foundation, in* 1751, *to* 1800, *with remarks on the mode of treatment, &c.* by J. M. Wallace, M. D., *Resident Surgeon.*

Abstract of 197 cases, treated from 1751 to 1800.

| Description of Fracture. | Whole number admitted. | Whole number cured. | Number of days required for cure. Maximum. | Minimum. | Average. | Number of days in which greatest proportion were cured. | Died from immediate effects of injury. | Died Subsequently. | Terminated by amputation. | Removed while under treatment. |
|---|---|---|---|---|---|---|---|---|---|---|
| Leg, Simple | 55 | 41 | 205 | 10 | 87 | 55 to 65 | 2 | 1 | 2 | 9 |
| " Compound | 8 | 7 | 201 | 53 | 104 | 80 to 90 | 0 | 0 | 1 | 0 |
| " Complicated | 9 | 9 | 591 | 77 | 213 | 180 to 190 | 0 | 0 | 0 | 0 |
| Thigh, Simple | 31 | 23 | 245 | 29 | 96 | 70 to 80 | 2 | 2 | 0 | 4 |
| " Compound | 1 | 1 | 199 | 199 | 199 | 199 | 0 | 0 | 0 | 0 |
| " Complicated | 10 | 10 | 314 | 42 | 118 | 90 to 100 | 0 | 0 | 0 | 0 |
| Arm, Simple | 34 | 24 | 162 | 21 | 69 | 35 to 45 | 3 | 0 | 0 | 7 |
| " Complicated | 2 | 2 | 123 | 32 | 77 | 32 to 123 | 0 | 0 | 0 | 0 |
| Ribs | 20 | 13 | 195 | 11 | 50 | 25 to 35 | 2 | 0 | 0 | 5 |
| Skull | 9 | 4 | 205 | 35 | 137 | 150 to 160 | 3 | 1 | 0 | 1 |
| Clavicle | 8 | 7 | 42 | 16 | 29 | 25 to 35 | 0 | 0 | 0 | 1 |
| Jaw | 6 | 4 | 192 | 40 | 91 | 50 to 60 | 1 | 0 | 0 | 1 |
| Patella | 3 | 3 | 151 | 79 | 114 | 79 to 151 | 0 | 0 | 0 | 0 |
| Vertebræ | 1 | 1 | 507 | 507 | 507 | 507 | 0 | 0 | 0 | 0 |
| Pelvis | 1 | 0 | 0 | 0 | 0 | 0 | 0 | 0 | 0 | 1 |
| Sternum | 1 | 1 | 10 | 10 | 10 | 10 | 0 | 0 | 0 | 0 |
| Wrist | 1 | 1 | 255 | 255 | 255 | 255 | 0 | 0 | 0 | 0 |
| Fingers | 1 | 1 | 31 | 31 | 31 | 31 | 0 | 0 | 0 | 0 |
| Toes | 1 | 1 | 26 | 26 | 26 | 26 | 0 | 0 | 0 | 0 |
| Not designated | 4 | 1 | 72 | 72 | 72 | 72 | 2 | 0 | 0 | 1 |

Abstract of 868 cases treated from 1800 to 1829.

| Description of Fracture. | Whole number admitted. | Whole number cured. | Number of days required for cure. Maximum. | Minimum. | Average. | Number of days in which greatest proportion were cured. | Died from immediate effects of injury. | Died Subsequently. | Terminated by amputation. | Removed while under treatment. |
|---|---|---|---|---|---|---|---|---|---|---|
| Leg, Simple | 249 | 216 | 217 | 10 | 70 | 50 to 60 | 9 | 12 | 0 | 12 |
| " Compound | 10 | 3 | 220 | 83 | 136 | 80 to 110 | 6 | 1 | 0 | 0 |
| " Complicated | 21 | 9 | 518 | 51 | 204 | 55 to 65 | 5 | 5 | 2 | 0 |
| Arm, Simple | 192 | 168 | 181 | 11 | 48 | 35 to 45 | 3 | 3 | 0 | 18 |
| " Complicated | 8 | 5 | 66 | 25 | 48 | 50 to 60 | 3 | 0 | 0 | 0 |
| Thigh, Simple | 130 | 106 | 210 | 32 | 110 | 65 to 75 | 8 | 3 | 0 | 13 |
| " Compound | 2 | 0 | 0 | 0 | 0 | 0 0 | 2 | 0 | 0 | 0 |
| " Complicated | 18 | 8 | 418 | 77 | 158 | 110 to 120 | 7 | 2 | 0 | 1 |
| Clavicle, Simple | 61 | 58 | 84 | 9 | 36 | 30 to 40 | 0 | 0 | 0 | 3 |
| " Complicated | 2 | 0 | 0 | 0 | 0 | 0 0 | 1 | 1 | 0 | 0 |
| Ribs, Simple | 45 | 38 | 70 | 6 | 34 | 20 to 30 | 2 | 0 | 0 | 5 |
| " Complicated | 7 | 2 | 70 | 43 | 56 | 43 to 70 | 5 | 0 | 0 | 0 |
| Skull | 29 | 9 | 133 | 10 | 50 | 40 to 50 | 16 | 1 | 0 | 3 |
| Jaw | 22 | 15 | 136 | 11 | 34 | 25 to 35 | 1 | 1 | 0 | 5 |
| Patella | 12 | 12 | 144 | 33 | 73 | 70 to 80 | 0 | 0 | 0 | 0 |
| Vertebræ | 10 | 0 | 0 | 0 | 0 | 0 0 | 2 | 7 | 0 | 1 |
| Fingers, Simple | 10 | 6 | 63 | 20 | 43 | 40 to 50 | 1 | 0 | 0 | 3 |
| " Compound | 1 | 1 | 74 | 74 | 74 | 74 | 0 | 0 | 0 | 0 |
| Elbow | 8 | 6 | 106 | 28 | 51 | 35 to 45 | 0 | 0 | 1 | 1 |
| Fibula | 6 | 5 | 175 | 51 | 99 | 100 to 120 | 0 | 1 | 0 | 0 |
| Nose | 4 | 2 | 22 | 9 | 15 | 9 to 22 | 0 | 0 | 0 | 2 |
| Shoulder | 4 | 3 | 58 | 26 | 39 | 25 to 35 | 0 | 0 | 0 | 1 |
| Scapula | 3 | 3 | 131 | 41 | 71 | 51 to 131 | 0 | 0 | 0 | 0 |
| Pelvis | 3 | 2 | 54 | 30 | 42 | 30 to 54 | 0 | 0 | 0 | 1 |
| Foot | 3 | 2 | 128 | 121 | 124 | 120 to 130 | 1 | 0 | 0 | 0 |
| Toes | 3 | 2 | 130 | 9 | 69 | 9 to 130 | 0 | 0 | 0 | 1 |
| Ankle | 3 | 2 | 66 | 42 | 54 | 42 to 66 | 1 | 0 | 0 | 0 |
| Hand | 2 | 2 | 48 | 23 | 35 | 23 to 48 | 0 | 0 | 0 | 0 |
| Not designated | 1 | 1 | 19 | 19 | 19 | 19 | 0 | 0 | 0 | 0 |

Abstract of 735 cases treated from 1829 to 1838.

| Description of Fracture. | Whole No. admitted. | Whole No. cured. | No. of days required for cure, in adults. Maximum. | Minimum. | Average. | No. of days in which greatest proportion were cured. | No. of days req'd for cure in children under 18 y'rs. Maximum. | Minimum. | Average. | No. of days in which greatest proportion were cured. | Died from the immediate effects of injury. | Died subsequently. | Terminated by amputation. | Removed while under treatment. |
|---|---|---|---|---|---|---|---|---|---|---|---|---|---|---|
| Leg, Simple | 172 | 150 | 516 | 14 | 74 | 55 to 65 | 88 | 44 | 64 | 55 to 65 | 11 | 5 | 1 | 5 |
| " Compound | 37 | 21 | 374 | 54 | 141 | 90 to 100 | 103 | 79 | 91 | 79 to 103 | 8 | 5 | 2 | 1 |
| " Complicated | 8 | 6 | 103 | 48 | 76 | 50 to 80 | 206 | 206 | 206 | 206 | 1 | 1 | 0 | 0 |
| Arm, Simple | 161 | 142 | 191 | 9 | 32 | 25 to 30 | 133 | 7 | 39 | 25 to 35 | 1 | 1 | 0 | 17 |
| " Compound | 11 | 4 | 381 | 17 | 156 | 17 to 381 | 54 | 54 | 54 | 54 | 4 | 2 | 1 | 0 |
| " Complicated | 10 | 6 | 81 | 42 | 54 | 45 to 55 | 0 | 0 | 0 | 00 | 2 | 0 | 1 | 1 |
| Clavical, Simple | 75 | 63 | 68 | 8 | 31 | 20 to 30 | 39 | 15 | 23 | 20 to 30 | 0 | 0 | 0 | 12 |
| " Complicated | 2 | 1 | 75 | 75 | 75 | 75 | 0 | 0 | 0 | 00 | 1 | 0 | 0 | 0 |
| Thigh, Simple | 73 | 60 | 254 | 40 | 92 | 65 to 75 | 137 | 14 | 66 | not fixed. | 7 | 0 | 0 | 6 |
| " Compound | 10 | 1 | 0 | 0 | 0 | 00 | 45 | 45 | 45 | 45 | 4 | 3 | 2 | 0 |
| " Complicated | 12 | 8 | 174 | 102 | 138 | 102 to 174 | 221 | 45 | 98 | 60 to 70 | 3 | 1 | 0 | 0 |
| Neck of thigh | 4 | 0 | 0 | 0 | 0 | 00 | 0 | 0 | 0 | 00 | 0 | 0 | 0 | 4 |

Abstract of 735 cases treated from 1829 to 1838—(continued.)

| Description of Fracture. | Whole No. admitted. | Whole No. cured. | No. of days required for cure, in adults. Maximum. | Minimum. | Average. | No. of days in which greatest proportion were cured. | No. of days req'd for cure in children under 18 y'rs. Maximum. | Minimum. | Average. | No. of days in which greatest proportion were cured. | Died from the immediate effects of the injury. | Died subsequently. | Terminated by amputation | Removed while under treatment. |
|---|---|---|---|---|---|---|---|---|---|---|---|---|---|---|
| SKULL, Simple | 31 | 13 | 156 | 14 | 57 | 20 to 60 | 115 | 18 | 66 | 18 to 115 | 15 | 1 | 0 | 2 |
| " Compound | 3 | 1 | 0 | 0 | 0 | 00 | 69 | 69 | 69 | 69 | 2 | 0 | 0 | 0 |
| " Complicated | 2 | 0 | 0 | 0 | 0 | 00 | 0 | 0 | 0 | 00 | 2 | 0 | 0 | 0 |
| RIBS, Simple | 35 | 29 | 116 | 4 | 29 | 20 to 30 | 0 | 0 | 0 | 00 | 3 | 1 | 0 | 2 |
| " Complicated | 3 | 2 | 72 | 21 | 46 | 21 to 72 | 0 | 0 | 0 | 00 | 1 | 0 | 0 | 0 |
| JAW, Simple | 17 | 11 | 58 | 17 | 38 | 30 to 40 | 126 | 33 | 79 | 33 to 126 | 4 | 1 | 0 | 1 |
| " Compound | 2 | 2 | 0 | 0 | 0 | 00 | 126 | 25 | 77 | 25 to 126 | 0 | 0 | 0 | 0 |
| PATELLA, Simple | 12 | 11 | 151 | 45 | 70 | 60 to 70 | 0 | 0 | 0 | 00 | 0 | 0 | 0 | 1 |
| " Compound | 1 | 1 | 140 | 140 | 140 | 140 | 0 | 0 | 0 | 00 | 0 | 0 | 0 | 0 |
| FINGERS, Simple | 8 | 4 | 108 | 17 | 52 | 17 to 108 | 0 | 0 | 0 | 00 | 1 | 0 | 2 | 1 |
| FIBULA, Simple | 6 | 5 | 47 | 25 | 33 | 25 to 35 | 0 | 0 | 0 | 00 | 0 | 0 | 0 | 1 |
| " Complicated | 1 | 1 | 73 | 73 | 73 | 73 | 0 | 0 | 0 | 00 | 0 | 0 | 0 | 0 |
| ELBOW, Simple | 8 | 8 | 74 | 19 | 51 | 40 to 50 | 44 | 37 | 40 | 37 to 44 | 0 | 0 | 0 | 0 |
| " Compound | 3 | 3 | 336 | 55 | 197 | 54 to 336 | 0 | 0 | 0 | 00 | 0 | 0 | 0 | 0 |
| SCAPULA, Simple | 6 | 4 | 106 | 16 | 46 | 35 to 45 | 0 | 0 | 0 | 00 | 0 | 1 | 0 | 1 |
| VERTEBRÆ | 4 | 0 | 0 | 0 | 0 | 00 | 0 | 0 | 0 | 00 | 1 | 3 | 0 | 0 |
| PELVIS, Compound | 1 | 0 | 0 | 0 | 0 | 00 | 0 | 0 | 0 | 00 | 1 | 0 | 0 | 0 |
| " ILIUM, simple | 3 | 3 | 113 | 34 | 62 | 34 to 113 | 0 | 0 | 0 | 00 | 0 | 0 | 0 | 0 |
| ANKLE, Simple | 2 | 1 | 39 | 39 | 39 | 39 | 0 | 0 | 0 | 00 | 0 | 0 | 0 | 1 |
| " Compound | 2 | 1 | 463 | 463 | 463 | 463 | 0 | 0 | 0 | 00 | 1 | 0 | 0 | 0 |
| STERNUM, Simple | 3 | 2 | 26 | 15 | 20 | 15 to 26 | 0 | 0 | 0 | 00 | 0 | 0 | 0 | 1 |
| NOSE, Simple | 2 | 2 | 19 | 15 | 17 | 15 to 19 | 0 | 0 | 0 | 00 | 0 | 0 | 0 | 0 |
| Not designated | 5 | 4 | 225 | 50 | 107 | 50 to 225 | 0 | 0 | 0 | 00 | 1 | 0 | 0 | 0 |

These three tables are formed from a record of all recent fractures, admitted since the year 1751, with the exception of nine, which were admitted during the last year, and are still under treatment. The records not having been kept by medical men, the kind of fractures is not so accurately specified as might be wished. Many cases, which were ascertained to be compound fractures, by a reference to the note books of the surgeons under whose charge they were, have been entered merely as "fractures." Fractures of the humerus, radius, and ulna, are all included under the term, fractured arm; and the expression, fracture of the leg, is used, without specifying whether one or both bones are implicated, except in a few instances. Up to the year 1829, the name of the patient, the fracture, its termination, together with the length of time occupied in the treatment, are all the details mentioned; in some cases, the accident is put down merely as a "fracture," without designating what part of the body was injured. In the annual accounts, which are laid before the contributors to the hospital, it has been the custom to embrace, under the general head of "fractures," all the cases, except those admitted for ununited fractures, and, on that account it has not been necessary to make an accurate distinction between them. In these statements, the cause of admission, assigned in the books, is the only one recognized, though it frequently happens that the patient is attacked with some other disease, which complicates very seriously his fracture, and, in fatal cases, the real cause of death is often different from that which appears in the hospital reports. Many patients, admitted for accidental injuries, are attacked with *mania a potu*, and in fractures this is particularly dangerous, from the impossibility of keeping the limb at rest during the violent exertions made under the influence of delirium. Should he recover from this disease, the violent inflammation produced by the frequent motion of the fragments on each other, frequently terminates in mortifiation and death. Another difficulty is the common occurrence of erysipelas. For many years, it has prevailed epidemically through the hospital, and, more especially, in the surgical wards. When puerperal fever was so common in the obstetrical ward, it was remarked, that a large majority of the surgical patients was attacked with erysipelas, and, in the medical wards and lunatic department, the slightest cause was sufficient to produce it. When compound fractures, or fractures attended with much inflammation, are admitted at these seasons, the treatment is more difficult, and the result frequently more unfavorable.

The class of patients admitted are, principally labourers and mechanics who support themselves by their daily work, and are therefore, unable to remain at home, without doing something towards their own maintenance. In a few cases, patients have preferred taking their discharge, as soon as they could safely leave the house, and have been permitted to go out, with the dressings still applied, and to return, occasionally, when they required adjusting. It is likely that some few cases, reported as cured, in a very short space of time, were of this description; as a general rule, however, they remain for some time after bony union has taken place, until the swelling and stiffness of the limb has subsided, and they can resume their usual occupations.

Previous to the year 1800, fractures of the lower extremities were very commonly treated with stiff pasteboard splints, wetted and moulded to the limb; after that time, Boyer's long splints, modified by Dr. Hartshorne were used, more especially in compound fractures, in consequence of the facility with which the dressings could be applied to the wound, without disturbing the limb. In simple fractures, after the disposition to muscular contraction was overcome by the application of these splints, a fracture box was generally used. Dr. Physick also introduced his improvement upon Dessault's splints, and, in most cases of fractured thigh, one or the other kind of apparatus was employed, while the fracture box came more and more into use when the leg was the part affected; for the last few years, the fractures of the thigh have been treated with Dessault's splints, while the fracture box is used, with scarcely an exception, in cases of the leg. The patient is placed on a firm mattress, with a hole in the middle for his fœces; a pillow, large enough to make some pressure on the leg, when the sides of the box are drawn together, is covered with an oil cloth and laid in the box. Upon this, the leg is adjusted, and the sides of the box tightened upon it; evaporating lotions are used, until the violence of the inflammation is subdued. The oil cloth is then removed, and when the bony union is tolerably firm, a roller is applied, and over that, pasteboard splints are adapted to the limb. In cases of compound fracture, during the summer season, the box is often filled with bran upon which the leg rests.

When there is a profuse discharge from the wound, the suspensory splints of Dr. N. R. Smith, of Baltimore, are found serviceable. By reference to the table, it will be seen, that but two cases of compound fracture of the thigh are reported, as having recovered, during this whole period of 86 years. It is possible, that cases of compound fracture have been admitted, when the fact was not specified. One of the cases cured came under my personal notice; it was a black child, four years old, admitted last summer, in whom, there was a separation of the diaphysis and epiphysis, at the lower part of the thigh. There was a small wound, four lines in length, through which the bone was *not* seen to protrude, nor was the wound probed; it afterwards united by the first intention. I hardly think, that there was here sufficient evidence of the existence of compound fracture: the other case, marked as cured, occurred in 1799. There is a patient now in the hospital, 18 years of age, admitted for this injury in June last. He is now able to move about the ward with the assistance of crutches; a large portion of the body of the femur is separating, and, after its removal, there is a probability of his recovery. Three cases, admitted within the last three years, terminated fatally, after being a long time in the hospital,—two from the formation of sloughs on the sacrum, and scapulæ, and from metastatic abscesses in the lungs; the other was worn out by profuse suppuration, and repeated attacks of erysipelas and diarrhœa.

The fractures of the humerus, below the insertion of the deltoid muscle, are here treated on a somewhat peculiar plan. An angular splint, reaching from the axilla down to the fingers, is applied, together with three short splints, as far as the elbow, and the whole secured by a roller. By these means, no motion can take place, except at the shoulder joint, and the fragments are kept in closer contact, than when four short splints are used.

In fractures of the clavicle, Dessault's apparatus was, for a long time, applied. A modification was suggested by Dr. Fox, resident surgeon in 1827, which obviates many inconveniences attending the use of Dessault's plan, and has since been used, to the exclusion of other methods of treatment. A pad, placed in the axilla, is secured by tapes, passing over to the shoulder of the opposite side, and a cap of brown Holland, cut to resemble the sleeve of a coat, open its whole length at the inner part, is applied from the wrist to four or five inches above the point of the elbow; loops are fastened to the ends of this, and bands passed from them to a circular collar, fixed on the shoulder of the sound side, draw the elbow down, and answer the purpose of the second bandage of Dessault.

